# Habitual food consumption of the Belgian population in 2014-2015 and adherence to food-based dietary guidelines

**DOI:** 10.1186/s13690-019-0343-3

**Published:** 2019-04-05

**Authors:** Sarah Bel, Karin A. A. De Ridder, Thérésa Lebacq, Cloë Ost, Eveline Teppers, Koenraad Cuypers, Jean Tafforeau

**Affiliations:** 1 Sciensano, Department of Epidemiology and public health, Unit Lifestyle and chronic diseases, Brussels, Belgium; 2 Centre de recherche en Epidémiologie, Biostatistiques et Recherches cliniques, Université libre de Bruxelles, Ecole de santé publique, Brussels, Belgium; 3 HIVA – Research Institute for Work and Society, Research Group Social and Economic Policy & Social Inclusion, Leuven, Belgium

**Keywords:** Habitual food consumption, survey, food-based dietary guidelines, 24-hour recall, children, adolescents, adults, Belgium

## Abstract

**Background:**

Between 2014 and 2015 a second National Food Consumption Survey was conducted in Belgium in order to evaluate the habitual food consumption in the general Belgian population and to compare it with food-based dietary guidelines (FBDG) and results of the 2004 Food Consumption Survey.

**Methods:**

A representative sample of the Belgian population was randomly selected from the National Population Register following a multistage stratified sampling procedure. Information on dietary intake was collected from 3146 subjects between 3 and 64 year old through two non-consecutive 24-hour dietary recalls using GloboDiet®. In addition, a self-administered food frequency questionnaire was completed. The distribution of habitual food consumptions and proportion of persons who did not meet the recommendations were estimated with SPADE.

**Results:**

For most of food groups analysed, the habitual consumption did not comply with FBDG. The consumption of nutrient-poor and energy-dense foods (e.g. alcohol, soft drinks and snacks) was excessive (35% of total energy intake), while the consumption of most other food groups was below the minimum recommended. A large majority of the population had an inadequate consumption of dairy products (98%), vegetables (95%), fruit (91%), potatoes, rice and pasta (88%) bread and cereals (83%) and water and sugar-free drinks (73%). Males had higher consumption of most food groups than females, thereby complying more often with FBDG. For all food groups, except dairy products and fruit, the consumption increased with age. The proportion of individuals meeting FBDG was the highest among young children (3-5 years) and the worst among adolescents aged 14-17 years old. Habitual consumption remained stable between 2004 and 2014 in the population aged 15-64 years old for all food groups except for increased consumption of water and sugar-free drinks (1180 to 1289 g/d) and decreased consumption of spreadable and cooking fat (27 to 19 g/d), red meat (34 to 25 g/d) and bread and cereals (173 to 142 g/d).

**Conclusions:**

The habitual food consumption of the Belgian population (3-64 years) in 2014-2015 deviates largely from FBDG, particularly among adolescents aged between 14 and 17 years old. Few improvements were observed between 2004 and 2014 in the population between 15 and 64 years old. Further efforts are therefore necessary to improve dietary habits in Belgium, in order to prevent and reduce diet-related diseases.

## Background

Dietary behaviours were ranked by the Global Burden of Disease as the third most important risk factor for death and disability in Belgium in 2017 [1]. To address this modifiable risk factor there is a need to develop and implement effective nutrition and food policies. Food consumption surveys provide useful insight into the consumption of foods and trends in dietary behaviour; they are also necessary to formulate and evaluate these policies, and can be used as a basis for education programmes and scientific research. The previous Belgian Food Consumption Survey was conducted in 2004 in the population above 15 years old (BNFCS 2004). In order to provide recent food consumption data and data for children and adolescents, a new Food Consumption Survey was conducted in 2014-2015 in the Belgian population between 3 and 64 year old (BNFCS2014). A specific objective of the BNFCS2014 was to evaluate the habitual food consumption in the general Belgian population, including children above 3 years old and adolescents and to compare these with national dietary guidelines.

Health promotion authorities in the Flemish and French-speaking Belgian communities have developed similar food-based dietary guidelines (FBDG) in the form of a food triangle and a food pyramid [2, 3]. The present paper summarises the habitual consumption of several food groups and compares it with the recommendations proposed in 2011 by the Flemish active food triangle, as it was published in the extensive national reports [4]. The active food triangle was selected because, contrary to the food pyramid, it describes the guidelines quantifiable in terms of weights or volumes. This triangle was first published in 1997 and underwent several minor modifications over the years when nutrient reference values were updated by the Superior Health Council of Belgium in 2009 [5] or according to new scientifically supported recommendations. The active food triangle consists of eight food groups: water; potatoes and cereal products; vegetables; fruit; dairy products and calcium-enriched soya products; meat, fish, eggs and substitutes; spreadable and cooking fat and nutrient-poor foods (e.g. sugared soft drinks, alcoholic drinks, chocolate and salty snacks). The larger the surface of the food group in the triangle is, the larger the recommended daily consumption of this food group is. The group of nutrient-poor foods is presented separately from the triangle as the consumption of these foods is not essential and should be limited [2]. Physical activity constitutes the base of the triangle, but is not discussed in the present paper.

The objective of the current paper is threefold: (1) to present the habitual food consumption in different subgroups of the Belgian population (defined by gender and age) in 2014-2015; (2) to compare these consumptions with food-based dietary guidelines (FBDG) to determine the proportion of the population that does not meet these recommendations; and (3) to compare these results with those of the Belgian National Food Consumption Survey of 2004 (BNFCS2004).

## Methods

The methodology of the BNFCS2014 followed to a large extent the guidelines published by the European Food Safety Authority (EFSA) in view of the EU Menu project [6, 7], as well as the recommendations made after the “Pilot Study for the Assessment of Nutrient intake and food Consumption Among Kids in Europe” (PANCAKE) project [8, 9]. The study was approved by the Ethical Committee of the University of Ghent and the Commission for the Privacy Protection. The study design and methodology of the BNFCS2014 and BNFCS2004 have been described in detail elsewhere [10, 11]. The BNFCS2004 had the same study design as the BNFCS2014, i.e. a multistage stratified sampling procedure with matched substitution in case of non-response or refusals, two non-consecutive face-to-face interviews (24-hour recalls) by dieticians with an earlier version of the GloboDiet® software and linkage to the NUBEL food composition database. As such, there is a high comparability between both surveys.

### Study design

A representative sample of the Belgian population aged 3 to 64 years was randomly selected from the National Population Register (reference year 2014) according to a multistage stratified sampling procedure, including: (1) a geographical stratification per province; (2) a selection of municipalities within each province; and (3) a selection of individuals within each municipality. A matched substitution was applied in case of a selected individual would not participate in the survey. The sample was stratified in ten strata defined by five age groups (3–5, 6–9, 10–17, 18–39, and 40–64 years) and both genders. Institutionalised persons, individuals unable to speak a national language and individuals physically or mentally unable to be interviewed were excluded.

In total, 9196 persons were invited to participate and ultimately 3461 persons participated. The final sample of this study contained data of 3146 participants because two 24-hour recall days were needed to calculate food consumption with SPADE. The participation rate (the ratio between the number of full participants, and the number of eligible and unresolved participants [6]) of the BNFCS2014 was only 37%, while the cooperation rate (the ratio between the number of full participants and the number of eligible participants [6]) was 43%.

Trained dieticians collected information from the participants through face-to-face interviews performed during two home visits with an interval from 2 to 4 weeks, between February 2014 and May 2015. In children (aged 3 to 9 years old) a parent or legal guardian was used as a proxy respondent. The data collection was distributed over the four seasons and all days of the week in order to incorporate seasonal effects and day-to-day variation in food intake. Since the data collection was extended with one trimester, relatively more interviews were performed in the winter (winter 43%, spring 18%, summer 15% and autumn 24%). 35% of the recall days were on Friday, Saturday or Sunday. Between the two visits, participants were asked to complete a food frequency questionnaire (FFQ) and a questionnaire about health. Children and adolescents were also asked to wear an accelerometer and fill in a logbook on physical activity.

### Collection of food consumption data

Food consumption data in adolescents and adults (10 to 64 years old) (n=2154) were collected using the 24-hour dietary recall method, carried out on two non-consecutive days. The interviewer asked the respondent to list all the foods, beverages and dietary supplements consumed in the preceding day, including their quantification. GloboDiet®, a computerised 24-hour recall program designed for the standardized collection of dietary data within a pan-European survey, was used as recommended by EFSA [6]. A new country-specific version of the software was prepared for this study (version 0.2014.02.10). GloboDiet® involves a structured and standardized approach to collect very detailed descriptions and quantities of consumed foods, recipes and dietary supplements. Food portion sizes were quantified using weights, volumes, shapes, thicknesses (e.g. spreads), household measures (e.g. glasses, cups, spoons, etc.), standard food portions (e.g. apples, packages) and a picture book including a selection of country-specific dishes in different portion sizes. These pictures were adapted from the EPIC-Soft picture book [12], the PANCAKE study picture book [13], the Swiss picture book [14] and the French picture book for the national survey of food consumption (INCA3) [15]. The picture book also included drawings of bread, representing the actual shape and size of bread slices, which were developed and used in the BNFCS2004 [16].

In children (3 to 9 years old) (n=992) dietary data were collected using two self-administered non-consecutive one-day food diaries followed by a GloboDiet® completion interview with the proxy respondent (parent or legal guardian): the first registration was made by telephone after the first home visit (or exceptionally by an additional face-to-face interview) and the second registration was conducted face-to-face at the respondent’s home during the second visit. The one-day food diaries had to be completed one or maximum two days before the completion interview with GloboDiet®. All food-diaries were open-ended (i.e., no pre-coded food lists) and special pages were available for detailing home-made recipes and dietary supplement intake. In every booklet explanations and examples on how to fill in the diary were provided.

All respondents were asked to self-report their usual frequency of consumption of 79 foods in the last 12 months (74 food items in children since alcoholic beverages were excluded). The frequency response options for each food item were: never, less than once per month, 1–3 times per month, once per week, 2–4 times per week, 5–6 times per week, once per day, 2–3 times per day and more than 3 times per day. The main objective of this food frequency questionnaire (FFQ) was to identify never-consumers (i.e., persons who indicate they never consume a specific food) for habitual consumption modelling purposes [6, 17].

Collected food consumption data were afterwards linked with detailed information on the energy and nutrient concentration of each specific reported food item, using the Belgian Food Composition Data NUBEL including branded foods [18]. The Dutch Food Composition Table (NEVO) was also used as reference when the information could not be found in the NUBEL database [19].

All consumed foods were assigned to the appropriate food group of the Flemish active food triangle by dieticians and the sum of the food groups covers the entire diet [2]. The habitual consumption of the different food groups was expressed in g/day, except for the nutrient-poor foods for which it was expressed in kcal/day [2]. For nutrient-poor foods, the contribution (in %) to the total energy intake was calculated as the ratio between the estimated intake of nutrient-poor foods in kcal/day and the estimated total energy intake in kcal/day for the specific age group. The habitual consumption was based on the edible part of the food and the portion size quantified as consumed weight (i.e. cooked if it was cooked). Food recipes were disaggregated into ingredients which could be registered as 1) ‘consumed’ or 2) with their ‘raw’ weight (before cooking the recipe). In case of the latter, raw weights were recalculated into consumed weights by using conversion factors. For the following food groups the daily recommendation is the minimum amount to reach: water and sugar free drinks; vegetables; fruit; bread and cereals; potatoes, rice and pasta; and dairy products and calcium enriched soya products. In case of a range, the lowest value has been set as the cut-off value. For the other food groups, meat, fish, eggs and substitutes; cheese; spreadable and cooking fat; and nutrient-poor foods, the recommended daily intake should not exceed the maximum value. The foods included in each category in the analyses are described in Table 1. A detailed description of this food selection can be found in the extensive web-based report (https://fcs.wiv-isp.be) [4]. Since the amount of spreadable fat in the recommendations was described as 5 gram per bread slice, the amount was recalculated into an age-specific amount depending on the number of recommended slices of bread for that age group. This amount was added to the standard amount of cooking fat and the total was used as a maximum value.

**Table 1:** Daily recommendations of the Flemish active food triangle in 2011, by age

		Age groups (years)				
Food group	Foods included in analyses	3-5	6-11	12-18	19-59	60+
Water and sugar-free drinks	Water=Water or aromatised water 0 kcal, Sugar-free drinks: tea and coffee without sugar, aromatised water or soft drinks if >0 and < 5 kcal/100g	500-1000 ml	1500 ml	1500 ml	1500 ml	1500 ml
Bread and cereals	Bread, breakfast cereals, oatmeal, rusks	90-175 g	150-315 g	210-420 g	210-420 g	150-315 g
Potatoes, rice and pasta	Potatoes, rice, pasta, quinoa, couscous, boulgour	50-200 g	210-280 g	240-350 g	240-350 g	210-280 g
Vegetables	Vegetables, tubers (excluding potatoes), vegetables in soup (>40% of the soup volume) and vegetable juice.	100-150 g	250-300 g	300 g	300 g	300 g
Fruit (including fruit juice and olives)	Fresh, dried, canned, compote, frozen fruit, fruit juice and olives.	100-200 g	250 g	375 g	250 g	250-375 g
Dairy products and calcium enriched soya products	(aromatised) milk, yoghurt, curd cheese, milk based desserts, calcium enriched drinks and desserts	500 ml	450 ml	600 ml	450 ml	600 ml
Cheese	All cheeses (except curd cheese)	20 g	20 g	40 g	20 g	40 g
Meat, fish, eggs and substitutes	Meat, fish and seafood: all, except fried. Eggs: all Substitutes: legumes, substitutes with >5 g protein/100 g Nuts and seeds	60 g	100 g	100 g	100 g	100 g
Spreadable* and cooking fat	All vegetable oils, animal fats, margarines and butter, including frying oil	Spreadable: 25 g Cooking: 15 g	Spreadable: 45 g Cooking: 15 g	Spreadable: 60 g Cooking: 15 g	Spreadable: 60 g Cooking: 15 g	Spreadable: 45 g Cooking: 15 g
Nutrient-poor foods (including alcohol)	Sugared soft drinks (> 5 kcal/ 100 g), sports drinks, energy drinks, cappuccino; alcoholic drinks (except if used in warm preparations); biscuits and pastries; confectionary and chocolates; ice cream; salt snacks, fried snacks; sauces	Less then 10% of total energy intake (110 kcal/day)	Less then 10% of total energy intake (145 kcal/day)	Less then 10% of total energy intake (230 kcal/day )	Less then 10% of total energy intake (250 kcal/day)	Less then 10% of total energy intake (250 kcal/day)

*The recommendation of spreadable fat is 5 g per slice of bread, the total recommended amount is calculated using the number of recommended slices of bread (e.g., for children between 3 and 5 years 3-5 slices of bread: 5*5 g = 25 g of spreadable fat)

Source: [2].

### Statistical analyses

The Statistical Program to Assess Dietary Exposure (SPADE) was used to estimate the habitual intake distribution of foods [20]. The repeated 24-hour recall and the FFQ data were combined to estimate the distribution of habitual food consumption based on statistical models that take into account a statistical correction for within-subject variation [17, 21]. SPADE is an R package which includes several modelling options, among which: (1) an option for foods that are consumed on a daily basis by almost all participants (1-part model); and (2) an option for foods that are consumed episodically (2-part model). This second model takes into account the information on never-consumers available for a specific food from the FFQ [20, 22]. The 2-part habitual intake is derived as the probability of consumption multiplied by the habitual intake amount on a consumption day, which are obtained by a Monte Carlo simulation and takes into account total residual variance and within- and between-person variances. Since these parameters might vary between subgroups of a certain group and the total group, the mean of the total group is not necessarily similar to the mean of the modelled means of its subgroups.

The distribution of habitual consumption was modelled as a function of age and this distribution was directly compared with the recommended age-specific consumption amount to estimate the proportion of individuals that did not comply with this cut-off value. Table 1 summarizes all dietary recommendations considered in this paper for the different food groups and age classes.

The analyses were weighted for age, gender, season and day of the week (i.e., week versus weekend) to ensure the results to be representative for the total Belgian population.

For Tables 2 to 5, 95% confidence intervals were provided by bootstrapping for the estimated food consumptions and for estimated proportions of the population with a consumption below a defined age-specific cut-off point value of the FBDG [20].

**Table 2:** Habitual consumption (mean and 95% CI) of major food groups (g/day) in 3 to 64-year old Belgian population, 2014-2015

		Age groups (years old)												ALL	
		3-5 (*n*=454)		6-9 (*n*=538)		10-13 (*n*=449)		14-17 (*n*=479)		18-39 (*n*=620)		40-64 (*n*=606)		3-64 (*n*=3146)	
Food group	Gender	Mean (95% CI)	% below cut-off point (95% CI)	Mean (95% CI)	% below cut-off point (95% CI)	Mean (95% CI)	% below cut-off point (95% CI)	Mean (95% CI)	% below cut-off point (95% CI)	Mean (95% CI)	% cut-off point (95% CI)	Mean (95% CI)	% below cut-off point (95% CI)	Mean (95% CI)	% below cut-off point (95% CI)
Water and sugar-free drinks^a^	M	512 (476-570)	57 (50-62)	644 (605-678)	97 (96-98)	767 (715-809)	93 (92-96)	886 (817-924)	89 (87-92)	1225 (1140-1284)	72 (69-77)	1356 (1276-1429)	65 (61-71)	1165 (1107-1207)	73 (70-76)
	F	464 (445-521)	63 (54-65)	620 (586-653)	98 (98-99)	767 (711-790)	96 (95-97)	893 (828-915)	91 (90-94)	1170 (1107-1231)	77 (73-81)	1379 (1327-1451)	63 (58-67)	1155 (1117-1199)	74 (71-76)
	All	489 (459-521)	60 (55-64)	613 (591-638)	98 (97-99)	739 (705-772)	95 (94-96)	848 (811-888)	92 (90-93)	1194 (1145-1238)	75 (72-77	1404 (1342-1449)	62 (59-66)	1168 (1132-1199)	73 (71-75)
Bread and cereals^a^	M	100 (94-114)	46 (34-52)	131 (122-137)	69 (64-75)	154 (140-159)	69 (66-76)	168 (151-172)	77 (75-85)	180 (162-190)	70 (65-79)	165 (155-180)	74 (66-79)	165 (157-172)	71 (67-76)
	F	91 (90-117)	54 (28-56)	115 (109-121)	82 (77-86)	123 (114-129)	86 (83-90)	124 (113-130)	96 (94-98)	122 (113-129)	96 (94-98)	113 (106-121)	96 (94-98)	117 (112-122)	93 (91-94)
	All	101 (95-110)	45 (38-51)	121 (118-128)	75 (70-77)	134 (129-142)	79 (75-81)	141 (136-148)	88 (85-90)	150 (142-156)	84 (82-88)	140 (131-145)	85 (83-89)	141 (135-145)	83 (81-85)
Potatoes, rice and pasta ^a^	M	96 (86-107)	16 (9-22)	135 (123-143)	88 (84-92)	160 (145-169)	83 (79-88)	171 (155-181)	82 (79-88)	176 (162-192)	81 (75-86)	153 (141-169)	87 (81-91-	160 (150-170)	81 (77-85)
	F	89 (81-99)	15 (8-21)	110 (104-120)	96 (94-98)	119 (111-127)	96 (94-98)	121 (112-130)	97 (95-99)	125 (117-139)	97 (93-98)	113 (105-126)	98 (95-99)	117 (113-126)	94 (92-95)
	All	91 (87-101)	18 (12-21)	120 (116-129)	92 (89-94)	138 (131-145)	90 (87-92)	146 (138-152)	90 (88-93)	152 (143-162)	88 (85-91)	132 (125-142)	93 (90-95	138 (133-146)	88 (85-89)
Vegetables^a^	M	82 (73-96)	72 (61-79)	94 (88-102)	99 (98-100)	103 (96-113)	99 (98-100)	111 (103-122)	99 (99-100)	133 (127-151)	98 (96-99)	156 (146-177)	96 (92-98)	135 (131-149)	97 (94-98)
	F	88 (76-101)	67 (57-76)	94 (89-102)	99 (98-100)	102 (95-111)	99 (98-100)	111 (101-120)	99 (99-100)	149 (132-156)	96 (95-98)	177 (161-191)	93 (89-96)	150 (139-157)	94 (92-96)
	All	91 (78-94)	65 (61-75)	96 (90-99)	99 (99-100)	103 (94-109)	99 (99-100)	110 (105-119)	99 (99-100)	143 (135-151)	97 (96-98)	172 (158-179)	94 (92-96)	145 (138-150)	95 (94-97)
Fruit (including fruit juices and olives)^a^	M	254 (220-271)	11 (8-18)	227 (213-249)	63 (57-68)	194 (178-206)	81 (78-85)	167 (149-179)	94 (91-97)	155 (137-169)	83 (79-88)	162 (149-186)	81 (74-85)	170 (156-183)	78 (74-82)
	F	224 (216-247)	11 (7-14)	210 (202-227)	68 (63-71)	189 (180-204)	84 (80-86)	173 (164-187)	95 (94-97)	166 (148-178)	81 (78-87)	182 (164-201)	77 (71-83)	180 (168-191)	77 (73-81)
	All	244 (228-257)	11 (8-13)	230 (211-236)	62 (60-68)	196 (185-202)	81 (79-84)	172 (162-181)	94 (92-96)	162 (146-170)	81 (79-86)	179 (163-190)	77 (74-82)	179 (167-185)	76 (75-80)
Fruit (excluding fruit juices and olives)^a^	M	139 (130-153)	37 (29-40)	119 (110-129)	93 (90-96)	92 (85-100)	97 (96-99)	74 (67-82)	100 (99-100)	80 (71-91)	96 (94-98)	115 (101-131)	92 (88-95)	99 (91-109)	92 (90-94)
	F	130 (118-139)	38 (32-44)	118 (110-124)	95 (94-97)	102 (95-108)	98 (97-99)	94 (85-100)	100 (100-100)	101 (91-109)	96 (95-98)	135 (124-151)	90 (86-93)	117 (109-125)	92 (89-93)
	All	136 (127-142)	36 (32-41)	120 (112-124)	94 (92-96)	99 (92-103)	98 (97-98)	85 (79-89)	100 (99-100)	92 (84-98)	96 (95-97)	128 (118-138)	90 (87-93)	110 (103-115)	91 (90-93)
Dairy products and calcium enriched soya products^a^	M	295 (278-344)	91 (83-93)	264 (251-284)	89 (86-91)	226 (209-244)	96 (93-97)	197 (175-213)	99 (98-100)	148 (130-160)	98 (97-99)	115 (106-137)	99 (99-100)	155 (147-168)	98 (97-94)
	F	279 (238-315)	93 (88-97)	218 (202-234)	96 (93-97)	176 (164-193)	99 (98-99)	152 (140-170)	100 (100-100)	140 (126-154)	99 (98-100)	149 (132-160)	99 (99-100)	157 (147-165)	99 (98-99)
	All	301 (264-321)	89 (87-94)	248 (231-254)	91 (90-94)	203 (189-214)	97 (96-98)	173 (159-185)	100 (99-100)	143 (132-155)	99 (98-99)	139 (123-146)	99 (99-100)	160 (149-163)	98 (98-99)
Cheese^b^	M	15 (12-17)	76 (69-85)	18 (16-21)	64 (54-69)	21 (19-24)	74 (65-78)	26 (23-29)	83 (77-90)	37 (32-41)	20 (16-31)	34 (31-39)	30 (23-38)	32 (29-34)	38 (34-43)
	F	14 (13-17)	79 (68-83)	19 (17-21)	60 (54-67)	21 (19-22)	71 (69-78)	23 (21-25)	89 s(84-92)	27 (25-31)	37 (28-45)	31 (28-34)	34 (31-46)	27 (25-29)	46 (41-51)
	All	15 (13-16)	76 (72-82)	19 (17-20)	62 (57-67)	22 (20-23)	70 (69-76)	25 (23-26)	85 (82-88)	32 (29-34)	28 (23-35)	33 (30-36)	32 (29-41)	30 (28-31)	41 (38-45)
Meat, fish, eggs and substitutes ^b^	M	93 (81-98)	17 (12-30)	122 (113-128)	33 (29-42)	138 (131-148)	20 (15-26)	150 (142-160)	14 (10-19)	170 (164-185)	7 (4-10)	189 (178-208)	3 (2-6)	168 (163-180)	9 (7-12)
	F	77 (75-98)	32 (11-33)	100 (93-105)	55 (49-62)	109 (101-113)	44 (40-54)	113 (103-117)	41 (35-52)	119 (110-126)	35 (27-43)	125 (118-135)	29 (20-35)	117 (112-124)	34 (28-39)
	All	89 (81-92)	22 (19-31)	112 (105-116)	43 (40-50)	124 (119-130)	33 29-37)	131 (127-138)	28 (23-31)	146 (140-155)	19 (14-22)	159 (147-164)	13 (11-18)	145 (138-149)	20 (17-23)
Spreadable and cooking fat^b^	M	10 (9-13)	100 (98-100)	13 (12-14)	100 (100-100)	15 (13-16)	100 (100-100)	16 (14-17)	100 (100-100)	21 (17-21)	99 (99-100)	27 (23-30)	98 (96-100)	22 (19-23)	99 (98-100)
	F	12 (9-12)	100 (99-100)	12 (11-12)	100 (100-100)	12 (11-13)	100 (100-100)	12 (11-13)	100 (100-100)	14 (13-16)	100 (100-100)	18 (17-20)	100 (100-100)	15 (14-16)	100 (100-100)
	All	10 (9-12)	100 (99-100)	12 (11-13)	100 (100-100)	13 (12-14)	100 (100-100)	14 (13-15)	100 (100-100)	18 (16-18)	100 (100-100)	22 (20-24)	99 (99-100)	18 (17-19)	100 (99-100)
Nutrient-poor foods (kcal/day)^b^	M	502 (461-527)	3 (2-4)	669 (648-704)	1 (1-2)	782 (756-819)	1 (1-2)	846 (813-884)	2 (1-3)	880 (830-922)	2 (1-3)	753 (706-800)	4 (2-6)	792 (759-825)	3 (2-4)
	F	463 (434-488)	1 (1-2)	570 (549-595)	1 (0-1)	617 (595-644)	2 (1-2)	630 (607-657)	3 (2-4)	586 (559-613)	5 (4-7)	467 (443-492)	14 (11-18)	536 (516-554)	8 (6-10)
	All	479 (461-505)	3 (2-4)	632 (608-647)	1 (1-2)	712 (686-728)	2 (1-2)	744 (717-764)	3 (2-4)	721 (697-752)	4 (3-5)	598 (573-625)	9 (7-11)	656 (639-676)	6 (5-7)

*M* Male, *F* Female

^a^% below the cut-off point value: based on a recommendation defined as “a minimum amount to reach” and to be understood as a % not meeting the recommendations

^b^% below the cut-off point value: based on a recommendation defined as a “a maximum amount not to exceed” and to be understood as a % meeting the recommendations

Source: [4]

**Table 3:** Habitual consumption (mean and 95% CI) of major food groups (g/day) in 15 to 64-years old Belgian population, 2004 versus 2014-2015

	Mean (95% CI)	% below recommendation (95% CI)	Mean (95% CI)	% below recommendation (95% CI)
	Year			
Food group	2004		2014-2015	
Water and sugar-free drinks^a^	1180 (1140-1244)	75 (70-77)	1289 (1249-1329)	68 (65-71)
Bread and cereals^a^	173 (167-179)	71 (68-74)	142 (136-148)	86 (83-89)
Potatoes, rice and pasta^a^	149 (143-157)	92 (88-94)	142 (135-149)	90 (88-94)
Vegetables^a^	167 (160-178)	95 (92-96)	157 (149-164)	96 (94-98)
Fruit (including fruit juices and olives)^a^	185 (169-192)	77 (74-82)	170 (160-182)	80 (76-83)
Fruit (excluding fruit juices and olives)^a^	113 (106-120)	95 (93-96)	108 (103-119)	94 (90-95)
Dairy products and calcium enriched soya products^a^	154 (143-166)	98 (97-99)	139 (132-150)	99 (98-99)
Cheese^b^	30 (28-32)	39 (41-49)	32 (30-34)	35 (32-40)
Meat, fish, eggs and substitutes^a^	159 (152-163)	15 (13-19)	149 (146-157)	17 (12-19)
Red meat	34 (31-37)	-	25 (22-27)	-
White meat	19 (17-21)	-	23 (21-25)	-
Processed meat	64 (60-67)	-	68 (64-72)	-
Spreadable and cooking fat^b^	27 (26-29)	96 (95-97)	19 (19-21)	100 (99-100)
Nutrient-poor foods (kcal/day)^b^	730 (720-781)	6 (3-6)	674 (687-740)	7 (4-7)

^a^% below the cut-off point value: based on a recommendation defined as “a minimum amount to reach” and to be understood as a % not meeting the recommendations

^b^% below the cut-off point value: based on a recommendation defined as a “a maximum amount not to exceed” and to be understood as a % meeting the recommendations

Source: [4]

**Table 4:** Habitual consumption (mean and 95% CI) of different types of bread (g/day) in the 3 to 64-year old Belgian population, 2014-2015

Age (years old)	White breads (*n*=3088)	Brown breads (*n*=3128)	Wholegrain breads (*n*=3071)
3-5	39 (36-42)	23 (19-27)	5 (3-7)
6-9	52 (49-55)	23 (20-25)	5 (4-6)
10-13	61 (58-64)	24 (21-26)	4 (3-6)
14-17	64 (61-68)	26 (23-29)	5 (3-6)
18-39	59 (56-65)	36 (31-40)	7 (5-10)
40-64	44 (40-48)	45 (40-50)	11 (8-13)
All (3-64 years old)	52 (49-55)	36 (34-40)	8 (7-9)

*n* = number of persons with two 24-hour recall interviews and valid FFQ information

**Table 5:** Habitual consumption (mean and 95% CI) of different types of meat (g/day) in the 3 to 64-year old Belgian population, 2014-2015

Age (years old)	Red meat (*n*=3099)	White meat (*n*=3109)	Processed meat (*n*=3138)
3-5	8 (7-10)	13 (12-18)	45 (42-48)
6-9	12 (11-14)	18 (15-20)	56 (54-60)
10-13	15 (13-17)	20 (17-22)	63 (60-67)
14-17	19 (16-21)	22 (18-24)	67 (65-72)
18-39	22 (19-25)	24 (21-27)	70 (67-77)
40-64	29 (25-33)	23 (19-27)	63 (59-69)
ALL (3-64 years old)	23 (21-25)	22 (20-24)	65 (62-69)

*n* = number of persons with two 24-hour recall interviews and valid FFQ information

Since another statistical software was used in 2004, the data of BNFCS2004 were reanalysed with SPADE to ensure comparability between the results of the two surveys. In the present results, the habitual food consumption and compliance of the population with the recommendations were compared for the ages between 15 and 64 years only, as these were included in both surveys.

It was impossible to perform significance tests on the consumption results generated by SPADE to compare different subgroups in the population. The calculated amounts are the results of a multi-step Monte Carlo simulation model and as such it was not possible to apply non-parametric statistical tests on the skewed data. Therefore, the 95% confidence intervals were used to make such a comparison. When the confidence intervals were not overlapping, the consumptions were considered significantly different. Inversely, when the confidence intervals were overlapping, it was not possible to draw any conclusion. This approach can be considered as conservative, because some statistical significant differences might not have been identified.

## Results

### Study population

The demographic characteristics of our sample show that about 60% obtained a diploma of short- or long-type higher education, while this was only about 30% in the general Belgian population in 2014-2015 [23]. As weighting factors were used during the analysis, the results are representative for age, gender and province. The percentage of smokers in the current study population above 10 years old (24%) is similar to the percentage observed in the Belgian Health Interview Survey of 2013 (23% of the population 15 years and older) [24].

### Water and sugar-free drinks

The mean habitual consumption of water and sugar-free drinks (including tea, coffee and sugar-free soft drinks) was 1168 ml/day (95%CI 1132-1199) in the global 3-64 years old population. This consumption increased with age, but did not differ according to gender (Table 2). The Flemish food triangle recommends a daily consumption of 1500 ml of water and sugar-free drinks in individuals over 6 years old (500-1000 ml for the youngest children) (Table 1). The consumption level of the majority of the Belgian population (73%) was below this recommendation. The percentage of the individuals who did not meet the recommendations was lower in the 3-5 years group than in the older age groups. Almost all children and adolescents between 6 and 17 years old did not drink enough water or sugar-free drinks, especially the girls. This percentage was lower among adults, but still remains higher than 60%. A small but significant increase in the consumption of water and sugar-free drinks was observed in the 15-64 year old population between 2004 and 2014 (from 1180 to 1289 ml/day) (Table 3), due to an increase in the consumption of water (from 635 to 820 ml/day), despite a decrease in the consumption of sugar-free drinks (from 475 to 420 ml/day) (data not shown).

### Bread and cereals

The mean habitual consumption of bread and cereals (e.g., breakfast cereals, oatmeal, rusks) increased with age from the youngest children (3-5 years) to the younger adults (18-39 years) and decreased then in the older adults (40-64 years) (Table 2). After the age of 6, a large majority of the Belgian population (83%) was below the age-specific recommendations for this food group. The youngest children met the recommendation for bread and cereals more often (about 50%). As males had a significantly higher habitual consumption of bread and cereals than females, they also met the guidelines more frequently (Table 2). The habitual consumption of bread and cereals and the percentage of the 15-64 years old population reaching the recommendations decreased significantly between 2004 and 2014 (Table 3).

As a sensitivity analysis, the contribution of bread and cereals to the total energy intake was calculated by age and gender subgroups and no significant differences were observed.

An in-depth analysis of the consumption of different types of bread (white, wholegrain and brown bread) revealed that the consumption of white bread was the highest, followed by brown (i.e., between white and wholegrain bread) and wholegrain bread (Table 4). In Belgium bread and other bakery products can only be considered as wholemeal if it is exclusively made from 100% wholemeal flour. On the one hand, the consumption of white bread increased with age from 3-5 years until 14-17 years and decreased then until 40-64 years. On the other hand, the consumption of wholegrain bread and brown bread increased with age (Table 4).

### Potatoes, rice and pasta

The mean habitual consumption of potatoes and substitutes (e.g., rice or pasta) increased with age from 3-5 years to 18-39 years and then slightly decreased in older adults (Table 2). In age groups between 6 and 64 years, the majority of the Belgian population (about 90%) did not consume the age-specific recommended amounts, especially the girls. Among the youngest children (3-5 years) only 15% did not comply with the recommendations. As males had significantly higher habitual consumptions of potatoes and substitutes than females, they met these guidelines more frequently (19% versus 6%) (Table 2). The habitual consumption of potatoes and substitutes remained the same between both BNFCS2004 and BNFCS2014 (Table 3). Between both studies, the consumption of potatoes decreased, while the consumption of substitutes (e.g., pasta, rice) increased (data not shown).

### Vegetables

The mean habitual consumption of vegetables increased with age and did not differ according to gender (Table 2). More than 90% of the Belgian population above 6 years old did not meet the recommendation. This percentage was lower among the 3-5 years old children (67-72%). There were no significant differences between 2004 and 2014 in the consumption of vegetables (Table 3).

### Fruit

The mean habitual consumption of fruit (including fruit juice and olives) decreased with age from the youngest children (3-5 years) to the younger adults (18-39 years) and then slightly increased in older adults (40-64 years) (Table 2). Almost all (89%) children between 3 and 5 years old met their daily recommendation of 100 g of fruit (including fruit juices), but this proportion was lower (62-63%) when only the intake of fresh fruit was considered. Compared to the 3-5 years old, the percentage of the Belgian population consuming the recommended intake of fruit was lower in older children (37% in 6-9 years old) and in adolescents, with a minimum in adolescents between 14 and 17 years old (6%). In adults, about 20% of the population consumed enough fruits with respect to the recommendations. There were no significant differences between males and females in the consumption of fruit (Table 2). The consumption of fruit also remained the same between 2004 and 2014 (Table 3). When fruit juices and olives were not taken into account, a more U-form shaped age trend was observed: the youngest children (3-5 years old) had a mean consumption of 136 g/d, the adolescents of 14-17 years old had the lowest consumption of 85 g/d and the consumption increased to 128 g/d for the adults of 40-64 years old (Table 2).

### Dairy products and calcium enriched soya products

The mean habitual consumption of dairy and calcium enriched soya products (excluding cheese) decreased with age from 301 g/day in the youngest children to 141 g/day in the adults (Table 2). Between the age 6 and 17, boys consumed more dairy products than girls. Almost the whole Belgian population had a habitual consumption of these products below their age-specific recommendations (Table 2). The habitual consumption of dairy and calcium enriched soya products and the percentage reaching the recommendations were similar in 2004 and 2014 (Table 3).

### Cheese

The mean habitual consumption of cheese increased with age (Table 2), but was not significantly different by gender, except between the ages of 18 and 39 years old where males consumed more cheese than females, respectively 37 and 27 g/day. The percentage of the population which did not exceed the recommended amount was higher in females than in males and varied by age: 1) 76% of the boys and 79% of the girls (3-5 years old) did not exceed the recommended amount, 2) this percentage decreased among the children of 6-9 years (60% in males and 64% in females), 3) it increased again to 83% and 89% in the 14-17 years old male and female adolescents and 4) the percentage decreased to 30% in the adult population. The habitual consumption of cheese and the percentage within the recommendations were similar in 2004 and 2014 (Table 3).

### Meat, fish, eggs and substitutes

The mean habitual consumption of meat, fish, eggs and substitutes (e.g., legumes, tofu, tempeh, wheat gluten, nuts and seeds) increased with age (Table 2). Males consumed higher amounts of foods from this group than females (except in the youngest age group of 3-5 years old) and exceeded therefore more frequently the maximum recommended amounts. The percentage of the population that did not exceed the guidelines varied by age: 17% and 32% in 3-5 years old girls and boys, which increased to 33% and 55% in 6-9 years old girls and boys. It decreased again to 3% and 29% in 40-64 years old females and males. The habitual consumption of this food group and the percentage not exceeding the recommendations were similar in 2004 and 2014 (Table 3).

An in-depth analysis of different types of meat (i.e., red, white or processed) revealed that the consumption of processed meat was the highest in all age groups (65 g/day) (Table 5) and in males. In all age groups the habitual consumption of white meat was also higher than red meat, except in older adults (40-64 years) for which the consumption of red meat (29 g/day) was higher than white meat (23 g/day) (Table 5). Among 15-64 years old, the consumption of red meat decreased between 2004 and 2014, while the consumption of processed and white meat did not change significantly.

Results from the FFQ revealed that only 13% of the total population consumed fish at least twice a week as recommended. Adolescents complied less often with this recommendation (9-10%), while adults the most (16%). The habitual consumption of plant-based meat substitutes (such as legumes, vegetarian meat products, nuts or seeds) was very low in the Belgian population (4 g/day) (data not shown).

### Spreadable and cooking fat

The mean habitual consumption of spreadable and cooking fat increased with age (Table 2). This increase with age was more pronounced in males, whereas in females, the intake was relatively stable (12 g/day) during childhood and adolescence and only increased in adulthood (14-18 g/day). Males had a significantly higher habitual consumption of spreadable and cooking fat than females, especially in the adult age groups of 18-39 years (21 versus 14 g/day) and 40-64 years (27 versus 18 g/day).

All age and gender groups had a habitual consumption of spreadable and cooking fat below the maximum recommendations, except for 1% of the 18-39 years and 2% of the 40-64 years males (Table 2). The habitual consumption of spreadable and cooking fat, and the percentage of the population exceeding the maximum recommendations, decreased significantly between 2004 and 2014 (27 versus 19 g/day and 3.8 versus 0.4%) (Table 3).

### Nutrient-poor foods (including alcohol)

**Fig. 1:**
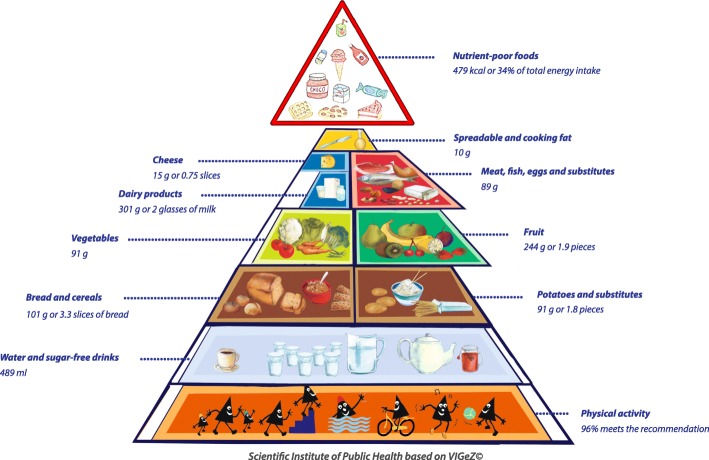
Compliance of the mean habitual food consumption with the food-based dietary guidelines in children (3-5 years)

**Fig. 2:**
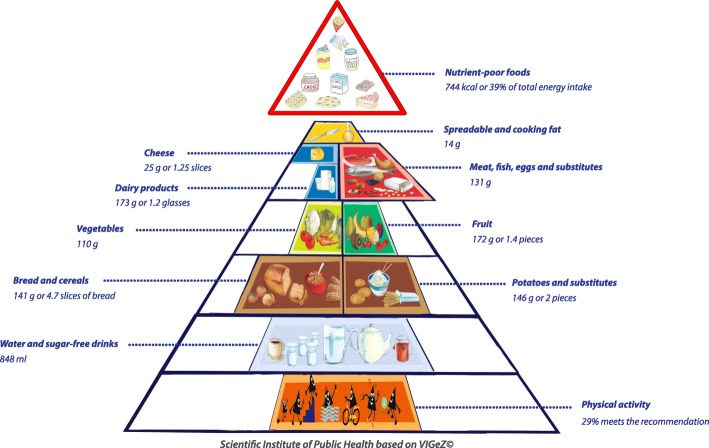
Compliance of the mean habitual food consumption with the food-based dietary guidelines in adolescents (14-17 years)

**Fig. 3:**
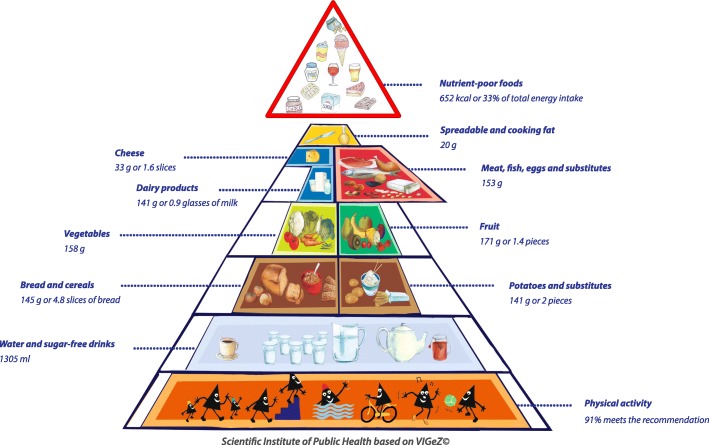
Compliance of the mean habitual food consumption with the food-based dietary guidelines in adults (18-64 years)

Only 6% of the Belgian population limited their energy intake from nutrient-poor foods (including alcohol) to 10% of their total energy intake, as recommended. Adolescents (10-17 years) and young adults (18-39 years) had the highest energy intake from this food group (Table 2). In the total population 35% of the total energy intake was contributed by nutrient-poor foods. In children (3-5 years old) the contribution of this food group to the total energy intake was 34% (Fig. 1), increased to 38% in the 6-9 years old group and remained at this level in adolescence (39%) (Fig. 2). During adulthood it decreased to 33% (Fig. 3). Women between 40 and 64 years met the recommendation the most frequently (14%) while males complied less often with these guidelines (Table 2). There were no significant differences in the habitual energy intake from nutrient-poor foods between the years 2004 and 2014 (Table 3).

### Graphic representation of adherence to FBDG

A graphic representation of the extent to which the 2014-2015 mean habitual food consumption deviated from the recommendations of the active food triangle is presented in Figs. 1 to 3. In each food group the compliance of the mean habitual intake with the recommended daily amount was presented by filling a certain percentage of the surface. For instance, if half of the surface was coloured, the habitual food consumption was half of the minimal recommended amount. Inversely, if the coloured surface went outside the borders of the triangle, the habitual consumption was higher than the recommended amount. The compliance to the guidelines was at its best among the youngest children of 3 to 5 years old (Fig. 1), but was worst for the adolescents between 14 to 17 years old (Fig. 2) and had again some improvement among the adults (Fig. 3).

## Discussion

### Main results

Overall, in 2014-2015, the habitual food consumption in the Belgium population between 3 and 64 years old did not comply with the FBDG as defined by the Active Food Triangle in 2011. The consumption of nutrient-poor and energy-dense foods was excessive as it contributed to more than one third of the total energy intake instead of maximum 10%. In addition, the habitual consumption of meat, fish, eggs (and substitutes) and cheese exceeded the age-specific recommendations. On the other hand, the habitual consumptions of water, cereal products, potatoes and substitutes, vegetables, fruits (excluding fruit juices and olives), and dairy products were below the age-specific recommended amounts. Concerning spreadable and cooking fat, compliance with the national guidelines was observed for a large part of the population. This is strongly related to the computation of the recommendation which results in quite high amounts (i.e., 5 g of spreadable fat per bread slice multiplied with the number bread slices recommended). More in-depth analyses of food groups like meat and bread showed a preference towards processed meat and white bread, which have been identified as more unhealthy food choices [25].

Males usually consumed larger amounts of bread and cereals; potatoes and substitutes; meat, fish, eggs and substitutes; cheese; spreadable and cooking fat; and nutrient-poor foods than females. Because the required energy intake in men is on average higher and the FBDG were not gender-specific, the proportion of the population complying with the recommendations was higher in males than in females for the group of cereal-based products, as well as for potatoes, rice and pasta. This hypothesis was confirmed by a sensitivity analysis showing that the energy contribution by bread and cereals was similar for all ages and gender. Further, the proportion of the population exceeding the maximum recommended amounts of meat and substitutes, cheese, spreadable and cooking fat, and nutrient-poor foods was higher in males than in females.

For most of food groups (i.e., water and sugar-free drinks, vegetables, cheese, meat, fish, eggs and substitutes, spreadable and cooking fat, bread and cereals, potatoes and substitutes, and nutrient-poor foods), the mean habitual consumption increased with age which can partly be explained by an increase in energy need between childhood and adulthood. However, the mean habitual consumption of dairy products decreased with age, which results in a consumption far below the FBDG. Reasons for such evolution in Switzerland have been linked to reduction of fat and cholesterol intake or to digestion difficulties [26]. The consumption of fruit decreased by age, reached its minimum in adolescence and increased again in adults. The adults aged 40-64 years old usually consumed smaller amounts of bread and cereals, potatoes and substitutes, and nutrient-poor foods than younger adults (18-39 years), but larger amounts of fruits. In general, the youngest children (3-5 years) had the best compliance with the FBDG. This is probably partly due to the lower daily recommended amounts specific for this age group, but also to higher parental control on the children’s diet. The proportion of individuals meeting the FBDG was lower in the two following age groups (6-9 and 10-13 years) and reached a minimum in adolescents between 14 and 17 years. Subsequently, adults complied a bit better with the guidelines than adolescents.

Overall, the habitual consumption of the major food groups did not differ between 2004 and 2014 in the population between 15 and 64 years old. Two positive changes were observed: an increase in the habitual consumption of water and sugar-free drinks, due to a significant increase in water consumption, and a decrease in the habitual consumption of spreadable and cooking fat. However, a decrease was observed in the habitual consumption of bread and cereals. It is in line with the decline observed internationally, where it was associated with a common misconception of dietary adequacy and low awareness of the amount that should be eaten for good health [27]. The consumption of potatoes has also decreased between 2004 and 2014, but as the consumption of rice and pasta has increased, the consumption of the total food group potatoes, rice and pasta has remained stable. Although the consumption of the protein group did not change significantly over time, it is positive to observe a reduction of the consumption of red meat. However, no changes were observed for the consumption of processed meat or white meat. In Australia, the total meat/poultry/fish consumption increased over time because of an increase in poultry and fish consumption and no change in the consumption of red meat and processed meat during a period of two decades [28].

### Comparison with other studies

A previous study conducted in 2008 and using the BNFCS2004 data established a substantial difference between the Flemish FBDG and usual food intake by the Belgian population in 2004 [29]. As observed in the current study, the consumption of food products from the energy-dense and nutrient-poor food group, meat and substitutes, and cheese was globally excessive while the consumption from all other groups, except for potatoes, rice and pasta, was below the recommendations [29].

Some comparisons can be made with the national food surveys of other European countries involved in the EU Menu Project (Netherlands, France and Germany) using a comparable methodology, i.e., collecting dietary intake data with two 24-hour recalls using GloboDiet® [6].

In the Netherlands a Food Consumption Survey was organised between 2012 and 2016 among 1-79 years old (n=4313) [30]. The mean habitual consumption of vegetables, fruit, cheese, fats and oils in the Dutch population (131, 113, 33 and 22 g/day respectively) is quite comparable with the consumption in the Belgian population. However, in the Netherlands the population consumes about double the amount of dairy products (about 319 g/day excluding cheese) than the population in Belgium [30]. The consumption of potatoes and fats and oils in 9-69 years old decreased in comparison with the previous Dutch survey of 2007-2010, which is in line with our results. However, in contrary to our study, the Dutch Food Consumption survey also revealed a significant decrease in the consumption of dairy products and meat (products), an increase in the consumption of fruit (especially by children) and no change in the consumption of bread and cereals and rice or pasta [30].

The individual and national study on food consumption (INCA3) conducted in France between 2014 and 2015 among individuals between 0 and 79 years old (n=5855) observed in comparable age groups (4-6, 7-10, 11-14, 15-17, 18-44 and 45-64 years old) a lower average consumption of vegetables (range from 65 to 154 g/d) [31]. The French children (4-10 years), adolescents (11-17 years) and youngest adults (18-44 years) have a very similar average consumption of fruit (excluding fruit juices and olives) (range from 135 to 81 g/d). The older adults (45-64 years) have a higher consumption of fruit (average 170 g/d) [31].

Results of the last German National Nutrition Monitoring in 2012/2013 in 14-80 years old (n=1840) reveal, separately in men and women, comparable average consumption amounts of vegetables (136 g/d and 140 g/d respectively) and dairy products including cheese (195 g/d and 187g/d respectively), but a higher consumption of fruit (144 g/d and 179 g/d respectively), fats and oils (29 g/d and 20 g/d respectively) and water and sugar-free drinks (i.e. coffee and tea) (1826 g/d and 1903 g/d respectively) [32]. In line with the present study, an increase in consumption of water and coffee/tea was observed in comparison with 2005-2007. However, in contrast to this study, a decrease in fruit (products) and increase in animal fat was observed [32].

At the European level, the HELENA-study among more than 3000 adolescents (12.5-17.5 years) in 2006-2007 also provided evidence that food consumption (collected with two 24-hour recalls) in European adolescents is not optimal when compared to the different FBDG from Europe and the USA [33]. In particular, adolescents eat less than half of the recommended amount of fruit and vegetables and less than two-thirds of the recommended amount of dairy products; at the opposite they consume much more meat (and meat products), oils, fats and sweets than recommended [33]. In Belgium, the situation is quite comparable as adolescents eat less than half of the recommended amount of fruit, less than two-thirds of the recommended amounts of dairy products and only about two-thirds of the recommended amount of vegetables. The habitual consumption of meat, fish, eggs and protein substitutes is also higher than recommended. Adolescence is a challenging period, during which young people progressively take independence regarding their parents – thereby also regarding their parent food habits – while spending more time with their peers. Consequently, many adolescents are resistant to interventions they feel impede their independence [34] and are increasingly influenced by their peers [35].

### Strengths and limitations

The main strengths of this study are the use of national food consumption survey data representative for the Belgian population and the use of statistical modelling to calculate habitual intakes (i.e., removing within-person day to day variability) and to assess the percentage of the population below a certain recommended cut-off value. Additional, the design and chosen methods adhere to the EU Menu Project protocol, which was developed to harmonise the food consumption data collections in Europe and will allow better comparison with other European countries in the future [6].

The apparent differences between the habitual consumption and recommendations could be partly due to under-reporting of food intake. The percentage of under-reporting was estimated by comparing the reported energy intake with the presumed energy requirements using the Goldberg cut-off method [36], revised by Black [37–39]. Based on this method, 24% of the total study population under-reported their energy intake. Under-reporting was observed more frequently in females than in males (27% versus 21%), and in adolescents (36%) and adults (31%) than in children (5%) (whose food intake was proxy-reported). The degree of under-reporting by adults in this study is much higher than found in the adult population (18-79 years) of the French national food consumption survey (18%) [31] and the German national dietary surveys between 2005 and 2013 (ranged from 11 to 17%) (14-80 years) [32]. However, the under-reporting by adolescents and children in this study is comparable to that of the French adolescents between 15 and 17 years (40%) and children between 7 and 10 years old (5%). Under-reporting could be explained by the under-reporting of unhealthy and energy-dense foods or by inaccurate portion size estimates. The presence of under-reporting can lead to an underestimation of the intake of certain foods and to underestimation of the proportion of the population with an adequate or excess intake of certain foods.

A drawback of the age-specific FBDG is that the recommendations are the same for both genders while the average energy requirements differ according to gender [40]. As such, it creates a bias in the proportion of males and females that comply with the FBDG.

Although great efforts were made to select a representative sample of the Belgian population, the low participation rate (37%) might have introduced a sampling bias. The differences in characteristics between individuals that participated and those entering the study as a replacement, because the earlier contacted participant refused participation, was investigated. Adults were more likely to enter the study as a replacement (60%) than adolescents (51%) or children (44%). The mean age of persons that entered the study as a replacement was significantly higher (24 years, 95%CI 23-25 years) then those that did not (20 years, 95%CI 19-21 years). Persons that lived in the Brussels-Capital region (61%), West Flanders (60%) and Limburg (58%) were most likely to be a replacement while persons that lived in Namur (46%), Flemish Brabant (46%) and Luxembourg (37%) the least. There was no significant difference in participation by gender. To ensure representativeness of the Belgian population, weighting factors (based on age, sex, province, season and weekday) were used. However, weighting for socioeconomic status was not possible and our sample was higher educated than the global Belgian population.

## Conclusion

The present study shows only marginal changes in the food consumption in Belgium compared with the previous food consumption survey of 2004. The habitual food consumption of the Belgian population, with differences by age and gender, still deviates largely from the FBDG. These results raise the need for further efforts in order to improve the dietary habits of the Belgian population, in particular in the adolescent age group, in order to prevent and reduce diet-related diseases. Regular monitoring of the habitual food consumption through regular food consumption surveys is important to evaluate the efforts implemented.

## Abbreviations

BNFCS2004: Belgian National Food Consumption Survey 2004
BNFCS2014: Belgian National Food Consumption Survey 2014-2015
EFSA: European Food Safety Authority
FBDG: Food-based dietary guidelines
FFQ: Food frequency questionnaire
SPADE: Statistical Program to Assess Dietary Exposure
